# Allelopathic compound 2-methoxy-1,4-naphthoquinone is broadly effective against pathogenic *Prototheca* species *in vitro* and *in vivo*

**DOI:** 10.1128/aac.00497-25

**Published:** 2025-08-27

**Authors:** Amir Aliramezani, Grzegorz Szewczyk, Krystian Mokrzyński, Izabela Ciastoń, Beatrycze Nowicka, Jakub M. Kwiecinski

**Affiliations:** 1 Department of Microbiology, Faculty of Biochemistry, Biophysics and Biotechnology, Jagiellonian University 37799, Kraków, Poland; 2 Department of Biophysics, Faculty of Biochemistry, Biophysics and Biotechnology, Jagiellonian University 37799, Kraków, Poland; 3 Department of Plant Physiology and Biochemistry, Faculty of Biochemistry, Biophysics and Biotechnology, Jagiellonian University 37799, Kraków, Poland; University of Iowa, Iowa City, Iowa, USA

**Keywords:** *Prototheca*, antimicrobial, allelopathy, naphthoquinones, 2-methoxy-1,4-naphthoquinone, biofilm, ROS, mouse model

## Abstract

Unpigmented, yeast-like *Prototheca* algae are emerging pathogens responsible for unusual infections in humans and animals. Their treatment is often unsuccessful, as traditional antifungals and antibacterials show unsatisfactory activity against *Prototheca*. Therefore, the development of improved drugs that consider the peculiarities of algal biology is needed. Here, we describe a novel anti-*Prototheca* compound, the 2-methoxy-1,4-naphthoquinone, identified among molecules known to mediate plant and algal allelopathy. The 2-methoxy-1,4-naphthoquinone was highly active *in vitro* against a range of *Prototheca* isolates from different species, both planktonically and in biofilms. Its mode of action included the induction of toxic reactive oxygen species, and it targeted energy generation in the pathogen’s mitochondria. It effectively treated *Prototheca bovis* skin infection in a mouse model, demonstrating translational potential. Altogether, this study not only identified a new effective anti-*Prototheca* compound, but also identified 1,4-naphthoquinone backbone as a lead structure for further therapeutic development and demonstrated the feasibility of exploring known anti-plant compounds as a source of anti-*Prototheca* treatments.

## INTRODUCTION

The genus *Prototheca* comprises a group of unusual yeast-like microbial pathogens. These unicellular, eukaryotic, unpigmented algae are the only plant species known to cause infections in vertebrate animals (1–3). The number of reported *Prototheca* infections and outbreaks is increasing each year, making it a concerning emerging pathogen (1, 2).

The most common type of *Prototheca* infection is bovine mastitis, which often presents as a chronic or subclinical disease, with elevated somatic cell counts and continuously decreasing milk yield (2–5). Although likely underdiagnosed, *Prototheca* is estimated to account for 13–16% of mastitis cases (6–9). No effective treatment is available, and infected cows must be culled, leading to significant economic losses (2, 5).

*Prototheca* infections in humans and companion animals (mainly dogs) are less common than in cows but can have severe consequences. In dogs, infections occur in otherwise healthy animals and typically present as enteric or disseminated disease and are almost always fatal despite treatment (4, 10, 11). In humans, localized (mainly cutaneous) infections are the most typical and usually occur in immunocompetent individuals. Disseminated and bloodstream infections are rarer and mainly affect immunocompromised patients. While localized infections are generally treatable (often with surgical intervention), with a success rate of about 70–80%, systemic and disseminated cases have a cure rate of only 30% and a mortality rate exceeding 50% (2, 12).

Bovine mastitis is predominantly caused by *P. bovis* (formerly *P. zopfii* genotype 2), with occasional involvement of *P. blaschkeae* (2, 4, 5). Infections in dogs and humans are mostly associated with *P. bovis* and *P. wickerhamii* (2, 11, 12). Additionally, *P. ciferrii* (formerly *P. zopfii* genotype 1), frequently found associated with cows and their environment (1), and previously considered non-pathogenic, may occasionally cause infections in humans and other animals (13, 14).

Current treatment of *Prototheca* infections relies on antifungal and antibacterial drugs, although only some of these agents show anti-algal activity (1, 2, 12, 15, 16). This limited efficacy likely stems from the biological differences between algae, fungi, and bacteria. The lack of effective drugs may explain the generally poor treatment outcomes. A more promising strategy would likely involve developing compounds that specifically exploit the unique plant-like biology of *Prototheca*. One such potential avenue is allelopathy—that is, a phenomenon in which secondary metabolites produced by one plant species inhibit the germination, growth, or survival of neighboring species. Allelopathy has been observed not only between higher plants, but also in interactions involving algae (17, 18). Among the best-studied allelopathic compounds are 1,4-naphthoquinones, which mediate numerous plant-plant and plant-microbe interactions (19, 20). This group of molecules, structurally derived from naphthalene (with a benzene ring connected to a conjugated cyclic 1,4-diketone), forms the basis of many bioactive compounds, including vitamin K, as well as various anti-cancer and anti-infectious agents (20). Notably, some 1,4-naphthoquinones have been shown to inhibit green algae, disturb their photosynthesis, and induce oxidative stress (21–24).

**Fig 1:**
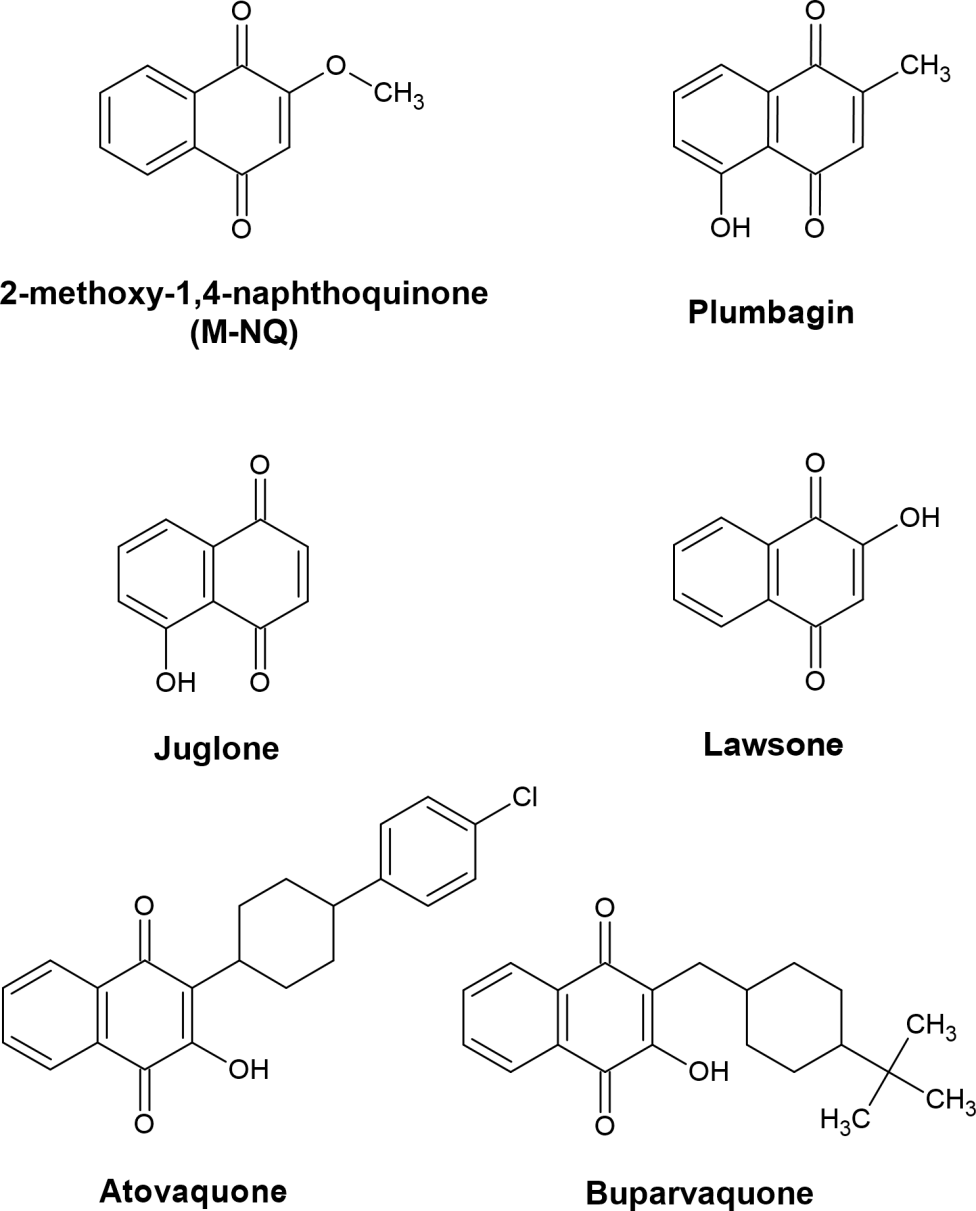
Chemical structures of 1,4-naphthoquinones examined in the study. M-NQ, plumbagin, juglone, and lawsone are naturally occurring plant allelopathic compounds. Atovaquone and buparvaquone are synthetic antiparasitic drugs based on the same 1,4-naphthoquinone backbone.

In this study, we investigated the anti-*Prototheca* potential of naturally occurring allelopathic 1,4-naphthoquinones, as well as selected synthetic drugs based on the 1,4-naphthoquinone scaffold (Fig. 1). We identified 2-methoxy-1,4-naphthoquinone (M-NQ, also known as lawsone methyl ether) as a highly potent anti-*Prototheca* compound. M-NQ was active against a broad range of isolates, in both planktonic and biofilm forms, and its action involved the induction of toxic reactive oxygen species (ROS) while disrupting energy generation in algal mitochondria. M-NQ also showed promising efficacy in an *in vivo* mouse infection model.

## RESULTS

### M-NQ has a potent anti-*Prototheca* activity *in vitro*

Different 1,4-naphthoquinones were tested for their anti-*Prototheca* activity, and M-NQ was identified as the most active, with minimum inhibitory concentration (MIC) of only 1 µg/mL against a broad range of *Prototheca* species (Table 1). Plumbagin showed several-fold lower activity than M-NQ. Juglone was even less effective, inhibiting the growth of only some strains and only at 16 µg/mL. Other compounds (lawsone, atovaquone, and buparvaquone) failed to inhibit *Prototheca* growth even at the highest tested concentration. When MIC and minimum cidal concentration (MCC) values were compared for the active compounds, MCCs were usually two- to fourfold higher than MICs, indicating that naphthoquinones have algicidal rather than merely algistatic properties.

**TABLE 1:** Minimum inhibitory concentrations (MICs) and minimum cidal concentrations (MCCs) of 1,4-naphthoquinones for *Prototheca* strains [µg/mL] measured with a microdilution method based on EUCAST

	M-NQ		Plumbagin		Juglone		Lawsone		Atovaquone		Buparvaquone	
	MIC	MCC	MIC	MCC	MIC	MCC	MIC	MCC	MIC	MCC	MIC	MCC
*P. bovis* O-2/22	1	4	8	16	16	16	>32	>32	>32	>32	>32	>32
*P. bovis* HKmikrobank1	1	4	8	8	16	16	>32	>32	>32	>32	>32	>32
*P. blaschkeae* IHEM 26958	1	2	4	8	>32	>32	>32	>32	>32	>32	>32	>32
*P. wickerhamii* CCUG 50031	1	2	16	32	>32	>32	>32	>32	>32	>32	>32	>32
*P. ciferrii* SAG 2063	1	2	2	4	16	16	>32	>32	>32	>32	>32	>32

To better characterize the anti-*Prototheca* potential of M-NQ, further experiments were performed using a representative strain *P. bovis* O-2/22, isolated from a large infectious outbreak (25).

**Fig 2:**
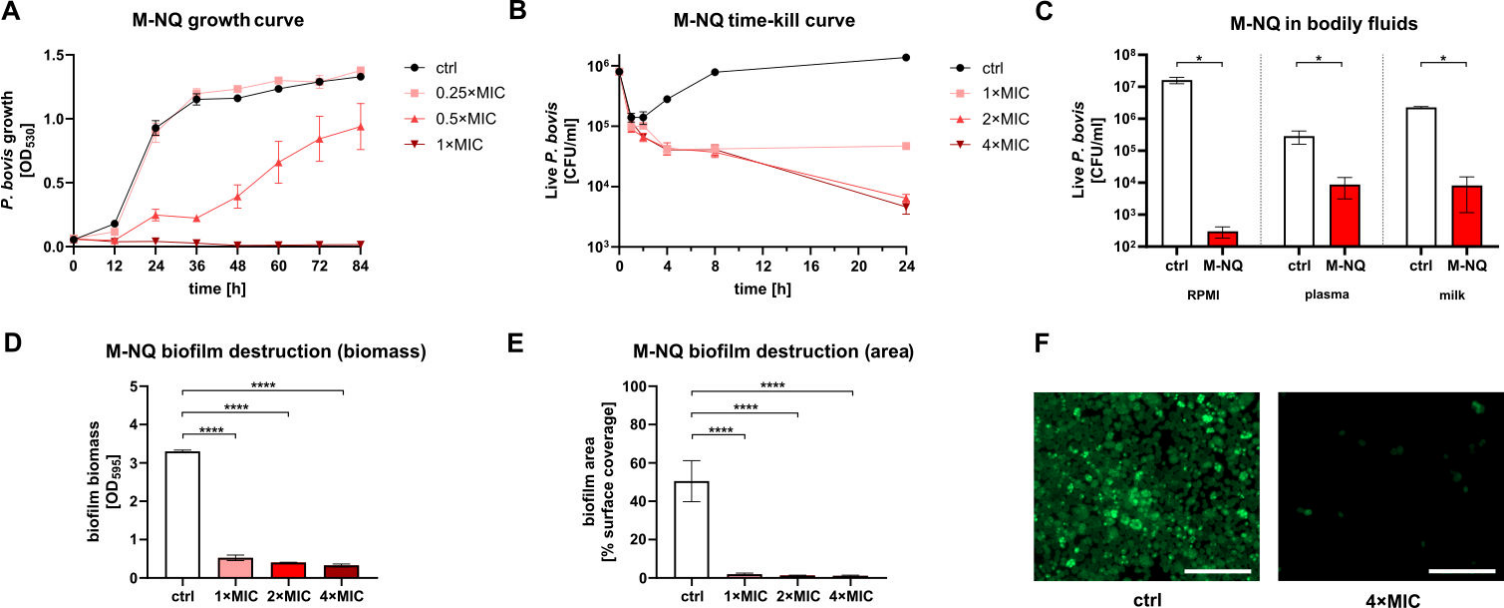
M-NQ is an effective anti-*Prototheca* compound. When added at sub-MIC concentrations to *P. bovis* cultures and incubated at 37°C, M-NQ reduced *P. bovis* growth, measured as OD_530_ turbidity increase (**A**). When added at 1× MIC or higher concentration to suspension of *P. bovis* in RPMI-2%G growth medium and incubated at 37°C, M-NQ induced killing of *P. bovis*, as evident in time-kill analysis with CFU counts (**B**). M-NQ retained its killing ability in bodily fluids when measured at 2× MIC after 24 h in the same setting as in the time-kill assay, but with microorganisms suspended in 50% human plasma or 50% cow milk in addition to RPMI-2%G (**C**). When added at 1× MIC or higher concentration to preformed 24-h-old biofilms of *P. bovis* and incubated for an additional 24 h at 37°C, M-NQ completely eliminated the biofilms. This was demonstrated by reduced biofilm biomass detected with crystal violet staining read at OD_595_ (**D**) and by reduced biofilm area coverage calculated from microscopic imaging of biofilms with *P. bovis* cells stained with Syto9 (**E and F**). Statistical significance was determined using the Mann-Whitney test (**C**) or ANOVA with Dunnett’s multiple comparison post-test (**D and E**); *P < 0.05; ****P < 0.0001. Data are shown as means ± SEM; *n* = 4 (**A–C**), *n* = 3 (**D**), and *n* = 10 (**E**). Representative fluorescent microscopy images of *P. bovis* biofilms are shown, scale bar = 100 µm (**F**). *P. bovis* O-2/22 was used for all assays.

In these detailed analyses, M-NQ showed good growth-inhibitory activity even at sub-MIC concentrations, with 0.5× MIC significantly delaying and reducing *P. bovis* growth (Fig. 2A). M-NQ also demonstrated strong cidal activity in a time-kill analysis. In control samples, after an initial drop in CFU caused by *P. bovis* strongly adhering to the tube walls, an increase in viable count was observed. In contrast, all drug-treated samples showed a continuing decrease in CFU, indicating time- and dose-dependent killing by M-NQ (Fig. 2B).

Importantly, M-NQ retained activity even under conditions mimicking infection sites, in the presence of potentially interfering bodily fluids. When tested in human blood plasma or cow milk, M-NQ exhibited slightly reduced activity compared to RPMI culture medium, but still effectively killed the pathogen (Fig. 2C).

### M-NQ is active against *Prototheca* biofilms

Biofilms, which may form during infection on implants or host tissues, are more resistant to antimicrobial agents than planktonic algal cells (26). However, M-NQ appeared highly effective even against biofilm-bound *Prototheca*. Addition of as little as 1× MIC of M-NQ to already established biofilms caused a dramatic decrease in biofilm biomass, with a slightly more pronounced reduction at higher concentrations (Fig. 2D). This pronounced biofilm-disrupting effect was also evident when biofilm area was quantified through fluorescent microscopy (Fig. 2E and F).

### M-NQ acts by inducing ROS and targeting energy generation

**Fig 3:**
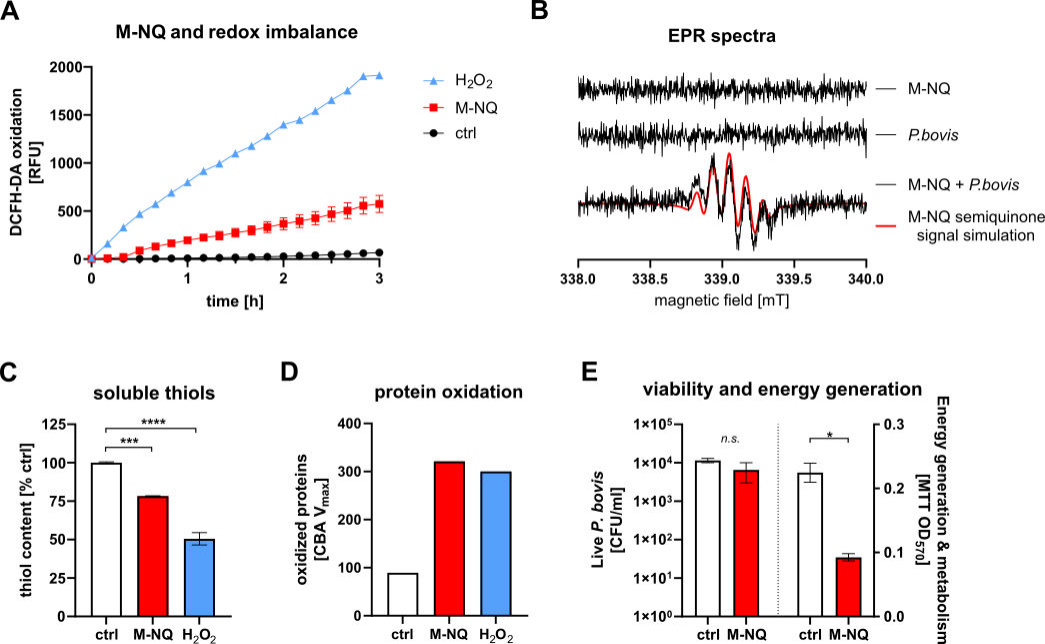
M-NQ induces ROS generation and damage to energy generation in mitochondria. When M-NQ was added at subinhibitory concentration (0.5× MIC) to a water suspension of *P. bovis* preloaded with the oxidation-sensitive probe DCFH-DA and incubated at 37°C, it shifted the intracellular redox balance towards more oxidative state, as indicated by increasing DCFH-DA fluorescence (**A**, RFU = relative fluorescence units). When EPR spectra were collected after ≈40 min at room temperature, they revealed the formation of a semiquinone radical in samples, where M-NQ was mixed at 200 µg/mL with *P. bovis* suspension in RPMI-2%G medium. This was detected as a characteristic signal in the spectra of the mixed sample, closely matching the simulated spectrum of the M-NQ semiquinone, absent in the spectra of M-NQ or *P. bovis* alone (**B**). Incubation of *P. bovis* with M-NQ (10 µg/mL) in RPMI-2%G was at 37°C for 3 h, followed by mechanical homogenization of the cells, revealed markers of oxidative stress: depletion of antioxidant thiols (measured using Ellman’s reagent and expressed as % of untreated control cells) (**C**), and formation of oxidized proteins (measured by increasing fluorescence of the CBA reagent and expressed as kinetic maximum speed of this increase, CBA *V*_max_) (**D**). After 2 h of incubation at 37°C with M-NQ at 1× MIC in RPMI-2%G, *P. bovis* cells showed a substantial loss of metabolic activity and energy generation (measured with MTT assay at OD_570_), indicating dysfunction of the electron transport chain in mitochondria. However, whole-cell viability (measured with CFU counts) remained unaffected, suggesting that the mitochondrial electron transport chain is targeted in early phases of M-NQ induced killing (**E**). Statistical significance was determined by the ANOVA with Dunnett’s multiple comparison post-test (**C and D**) or *t*-test (**E**); *P < 0.05; ***P < 0.001; ****P < 0.0001; *n.s*. not significant. Data shown as means ± SEM; *n* = 6 (**A**), *n* = 4 (**C**), and *n* = 2 (**E**). For (D), representative results from one experiment (out of four showing the same pattern) are shown. Positive ROS control of 50 nM H_2_O_2_ was used as indicated. *P. bovis* O-2/22 was used for all assays.

Some 1,4-naphthoquinones are thought to damage green algae through the generation of intracellular ROS (22, 23). Therefore, the ability of M-NQ to disrupt redox balance in live *Prototheca* was measured using a fluorescent redox-sensitive probe. At a subinhibitory dose (0.5× MIC), M-NQ induced a clear shift toward a more oxidative state inside *Prototheca*, as shown by intracellular oxidation of the probe (Fig. 3A). This response resembled the effect of the positive ROS control, H_2_O_2_, suggesting that M-NQ promotes ROS generation.

Electron paramagnetic resonance (EPR) spectroscopy was used to identify specific appearing ROS. EPR detected no radicals in M-NQ or *P. bovis* samples alone. However, mixing M-NQ with *P. bovis* produced a characteristic signal consistent with a simulated M-NQ semiquinone spectrum (Fig. 3B). This indicates that M-NQ is converted into its semiquinone radical form inside *Prototheca* cells, initiating redox cycling and promoting ROS formation. Additional evidence for ROS generation included M-NQ-induced depletion of antioxidant soluble thiols (Fig. 3C), and M-NQ-induced oxidative cellular damage, shown by an increase in oxidized cellular proteins (Fig. 3D). As thiol depletion could also result from M-NQ alkylating cysteine-rich cellular proteins, these results are not a proof of ROS induction being the only relevant mechanism of M-NQ action, but they do demonstrate that ROS generation is an important part of M-NQ’s action against *Prototheca*.

The most likely source of electrons required for intracellular M-NQ redox cycling is the mitochondrial respiratory electron transport chain. Therefore, mitochondria would not only be the sites of ROS generation, but electron interception would also block their respiratory activity. Consistent with this, exposure of *P. bovis* to M-NQ caused quick loss of metabolic activity and energy generation in mitochondrial electron transport chain, preceding the more generalized loss of whole-cell viability (Fig. 3E).

Taken together, these findings indicate that M-NQ exerts its toxic effects on *Prototheca* at least partly through ROS generation (via quinone redox cycling) and associated damage to mitochondrial energy generation, with possible additional contribution from M-NQ-induced alkylation of cellular proteins.

### M-NQ has some limited antibacterial activity

Since M-NQ and other quinones have been reported to have antibacterial activity (27–31), it was tested against a panel of bacteria associated with human and bovine infections. M-NQ displayed some activity against *Staphylococcus aureus*, and, to a lesser extent, against another gram-positive pathogen, *Streptococcus uberis* (Table 2). However, these effects were weaker than those observed against *Prototheca*. No activity was detected against gram-negative *Escherichia coli* or *Pseudomonas aeruginosa* (Table 2). This suggests that M-NQ is somewhat specific in its activity against *Prototheca*.

**TABLE 2:** Minimum inhibitory concentrations (MICs) and minimum cidal concentrations (MCCs) of M-NQ for bacterial strains [µg/mL] measured with a microdilution method based on EUCAST

	M-NQ	
	MIC	MCC
*S. aureus* Newman	4	4
*S. uberis* DSM 20569	16	>32
*E. coli* ATCC 25922	>32	>32
*P. aeruginosa* PAO1	>32	>32

### M-NQ has low toxicity to human cells

**Fig 4:**
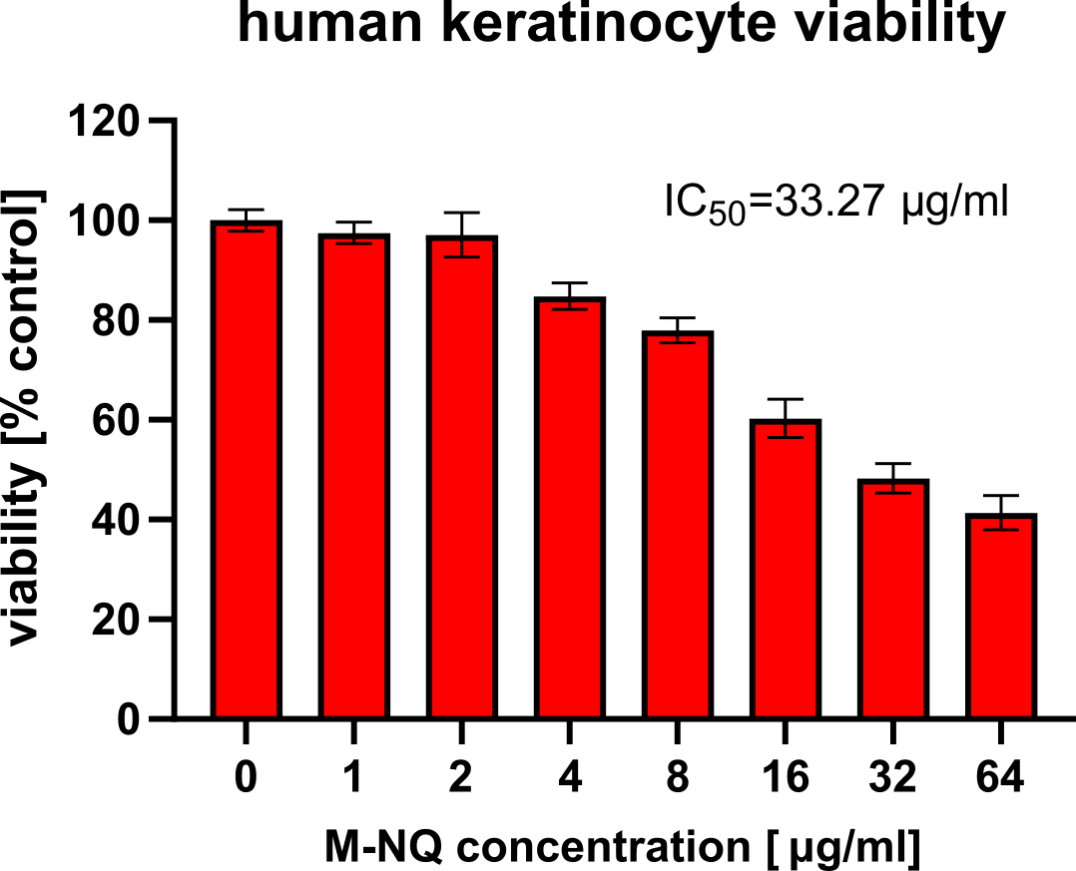
M-NQ has low toxicity to human cells. Viability of human HaCaT keratinocytes *in vitro* was measured with calcein AM staining after 24 h incubation with increasing doses of M-NQ. Data shown as means ± SEM, *n* = 4.

M-NQ has been reported to exhibit low toxicity in animal models and isolated human cells (30, 32). This was confirmed here in human HaCaT keratinocytes. While slight reductions in cell viability occurred at lower doses, only the highest tested concentration (>30-fold above the *Prototheca* MICs) reduced human cell viability by 50% (Fig. 4).

### M-NQ is effective *in vivo* in a mouse *Prototheca* infection model

**Fig 5:**
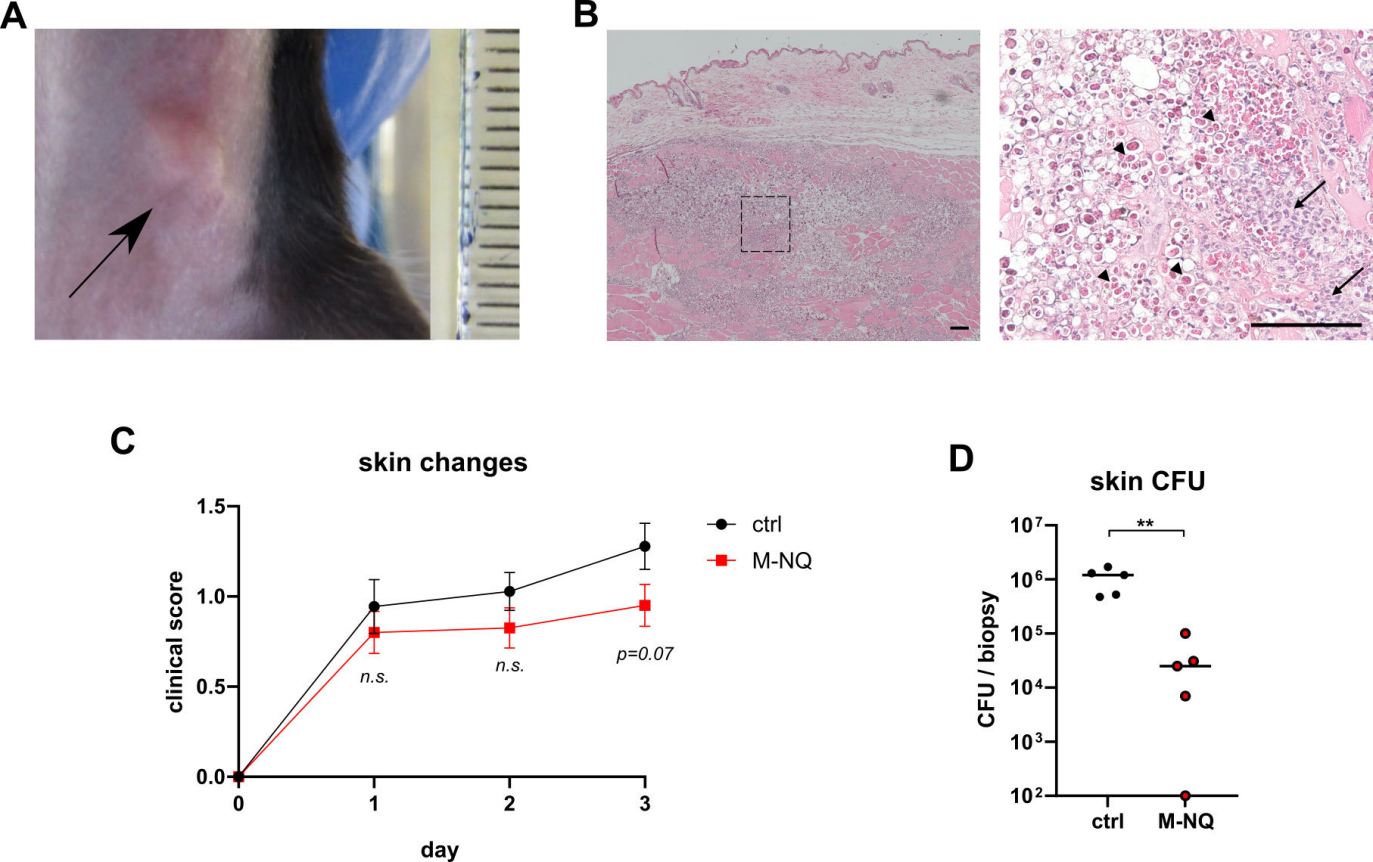
M-NQ is effective in mouse *P. bovis* infection model. Intradermal injection of *P. bovis* O-2/22 into transiently immunocompromised mice led to a development of a localized skin swelling at the infected site (**A**, arrow), characterized histologically by abundant *Prototheca* cells in subcutaneous tissues surrounded by a moderate inflammatory infiltrate (**B**, hematoxylin and eosin staining; arrows indicate infiltrating inflammatory cells; arrowheads mark *P. bovis* cells surrounded by characteristic unstained halos; scale bar = 100 µm). Oral treatment with M-NQ (15 mg/kg, twice per day), initiated 12 h post-infection, led to some reduction in visible skin changes (**C**) and significant decrease of *Prototheca* burden in the infected skin (**D**, detection limit: 1 × 10^2^ CFU/mL). Statistical significance was determined using the *t*-test (**C**) and Mann-Whitney test (**D**); **P < 0.01; *n.s*. not significant. Data shown as means ± SEM; *n* = 9-10 (**C**), *n* = 5 (**D**).

A therapeutic mouse model was established, based on the previously reported, well-tolerated M-NQ dosing of 15 mg/kg administered orally twice daily (30). Injection of *P. bovis* into the skin of transiently immunosuppressed mice produced localized infection, visible as subcutaneous swelling, and characterized histologically by abundant *Prototheca* in subcutaneous tissues, surrounded by sparse inflammatory infiltrates (Fig. 5A and B). When treatment with M-NQ was initiated 12 h after infection (when first symptoms became visible) and repeated every 12 h for a total of six doses, it influenced the infection course. Although the study timeframe was too short to observe clinical resolution, M-NQ-treated mice developed slightly milder symptoms (Fig. 5C). More importantly, treatment produced a significant two-log reduction in *Prototheca* burden in infected skin (Fig. 5D), which could possibly lead to clinical cure in a longer timeframe and which demonstrates the *in viv*o therapeutic potential of M-NQ.

## DISCUSSION

The lack of highly effective drugs has been an important obstacle in successfully treating *Prototheca* infections in humans and animals. Although the unique plant-like algal biology of *Prototheca* offers an intriguing target for drug development, this strategy remains largely unexplored. Moreover, previous similar attempts have mostly been unsuccessful. *Prototheca* are resistant to herbicide simazine (33), and compared to closely related green algae, they show greater resistance to herbicide aminotriazole (34, 35). Attempts to use plant hormones and their synthetic analogs yielded conflicting results, but even the most promising only partially inhibited *Prototheca* growth (33, 36, 37). Limited *in vitro* efficacy has so far been reported only for some dinitroaniline antimicrotubular agents and, at relatively high concentrations, for glyphosate (38, 39).

Our strategy, focusing on allelopathic 1,4-naphthoquinones, led to the successful identification of M-NQ as a potent anti-*Prototheca* agent. M-NQ completely inhibited growth of diverse *Prototheca* species at just 1 µg/mL, demonstrated direct algicidal activity, and retained effectiveness in bodily fluids likely to be present at infection sites, such as milk or blood plasma. Notably, M-NQ was highly effective against *Prototheca* biofilms, which are typically resistant to antimicrobial treatment (26). Concurrently, M-NQ appears to have a favorable safety profile for humans and other animals. In our *in vitro* human keratinocyte model, the concentration needed to decrease cell viability by half (IC_50_) was over 30 times higher than the MIC required to fully inhibit *Prototheca* growth. Even higher tolerances to M-NQ were reported in other human cell lines, with no toxicity observed at concentrations as high as 128 µg/mL (30). *In vivo*, oral administration of M-NQ at 15 mg/kg twice daily for 5 days caused no adverse effects in mice, and the acute toxic LD_50_ in an intraperitoneal injection is estimated at 71 mg/kg (30, 32), both doses well above effective anti-*Prototheca* concentrations. This combination of strong activity and relatively low toxicity suggests a clear translational potential for M-NQ as a treatment for *Prototheca* infections. Our proof-of-concept mouse model of *P. bovis* skin infection supports this. Although the experiment duration was likely too short to observe a clinical resolution, M-NQ reduced the *Prototheca* burden in infected tissue by nearly 99%, and in one case even fully cleared the infection, marking M-NQ as the first new experimental compound to demonstrate *in vivo* efficacy against *Prototheca*.

Antimicrobial 1,4-naphthoquinones have been reported to cause various types of damage to microbial cells, with disruption of cellular redox balance and generation of toxic ROS being a common mechanism (40). In the green microalgae *Chlamydomonas reinhardtii*, a relative of *Prototheca*, 1,4-naphthoquinone toxicity is mediated by redox cycling. 1,4-naphthoquinones intercept electrons from a photosynthetic electron transport chain, becoming reduced to highly reactive semiquinones and quinol dianions. These intermediates are spontaneously oxidized in the presence of oxygen, in the process generating ROS (superoxide, hydrogen peroxide), and regenerating the parent 1,4-naphthoquinone to repeat the cycle (22, 23). Our findings in *Prototheca* support a similar mechanism. M-NQ exposure in *Prototheca* caused a shift toward a more oxidized intracellular state, appearance of semiquinone radicals, and development of hallmarks of ROS-induced oxidative damage. Since *Prototheca* lack photosynthetic pigments, the likely source of electrons for M-NQ redox cycling is the mitochondrial respiratory electron transport chain—consistent with the suggestion of similar process in green algae growing with 1,4-naphthoquinones in the dark (23), and with direct observations in isolated animal mitochondria exposed to 1,4-naphthoquinones (41). This hypothesis is further supported by our observation that mitochondrial energy generation is among the earliest *Prototheca* cellular functions impaired by M-NQ exposure.

Other potential antimicrobial mechanisms of 1,4-naphthoquinones include chelation of intracellular iron and disruption of membrane integrity (40). Our preliminary experiments showed no effect of M-NQ on *Prototheca* membrane permeability (data not shown), and M-NQ lacks the large lipophilic tail of membrane-disrupting quinones (42), but metal chelation as a complementary mechanism alongside ROS induction deserves further investigation.

In studies of cancer cells, 1,4-naphthoquinones are known not only for ROS generation, but also for alkylation of cysteine-rich cellular proteins (43). The combined ROS- and alkylation-induced stress, along with possible direct inhibition of topoisomerases, interferes with the regulation of cell cycle, apoptosis, and intracellular signaling in the naphthoquinone-treated cells (43–45). M-NQ inside animal cells is thought to both undergo redox cycling and alkylate proteins (43), suggesting that its anti-*Prototheca* effect might also arise from a combination of ROS-induced oxidative damage and protein alkylation. Therefore, some of the observed changes (e.g., depletion of cellular thiols) probably result from both oxidation and alkylation. Given that *Prototheca* metabolism and cell regulatory pathways remain poorly understood, the full extent of these effects is not yet clear. Further study of *Prototheca* cellular biology will be critical for optimizing both 1,4-naphthoquinone-based therapeutics and all other types of novel anti-*Prototheca* agents.

A notable aspect of the tested 1,4-naphthoquinones is their specificity. While atovaquone and buparvaquone are effective against unicellular protozoan parasites (46), they showed no activity against *Prototheca*. In green *Chlamydomonas* algae, juglone is more toxic than plumbagin and M-NQ (22), the exact opposite of the pattern observed in the nonpigmented *Prototheca* algae. The variable inhibitory concentrations of M-NQ across bacterial species (27–31), confirmed by our observations, also point to fairly selective activity. Interestingly, although ROS induction and alkylation are the suggested mechanisms of its action, M-NQ is not the most potent ROS inducer or alkylator among the tested compounds, whereas juglone, a strong ROS inducer and alkylator, showed only moderate anti-*Prototheca* activity. This implies complex mechanisms of 1,4-naphthoquinone specificity, extending beyond their basic chemical reactivity. Such specificity could result from a combination of several properties. One factor might be the ability of compounds to cross diverse cellular barriers (e.g., outer membranes, cell walls, intracellular organellar membranes), which vary greatly between gram-positive and gram-negative bacteria, fungi, animal cells, and *Prototheca* algae. Additionally, distinct localization of targets (e.g., electron transport chains in bacterial membranes versus in mitochondria or chloroplasts in eukaryotes) requires different compound affinities for intracellular compartments to reach these targets. Differences in cellular metabolism, such as the presence of enzymes capable of reducing or oxidizing particular 1,4-naphthoquinones, or baseline levels of ROS and antioxidant defenses, may also play a role. While the mechanisms of 1,4-naphthoquinones specificity are not yet sufficiently understood for rational drug design, it is clear that different structural fragments of 1,4-naphthoquinone molecules are responsible for each of its chemical properties and cellular affinities (40, 44, 46). Ongoing research into 1,4-naphthoquinone specificity will aid future design of anti-*Prototheca* agents, but the inverse is also true: studies of quinones in *Prototheca*, with its unique cell structure and metabolism, can provide valuable insights into this class of compounds.

In summary, by focusing on allelopathic molecules, we have identified M-NQ as a new potent anti-*Prototheca* compound active against a broad spectrum of species, effective against both planktonic cells and in biofilms, and capable of treating *Prototheca* infections in a mouse model. We propose that its mechanism involves the induction of excessive ROS, and possibly protein alkylation, leading to loss of mitochondrial energy generation and cell death. These findings lay the groundwork for further development of M-NQ and related derivatives of the 1,4-naphthoquinone scaffold into novel therapeutics for *Prototheca* infections. Finally, our results validate the strategy of searching for anti-*Prototheca* compounds guided by understanding peculiarities of algal plant-like biology, rather than merely testing existing antimicrobials already developed against bacteria or fungi. This more focused approach holds promise for finally identifying effective treatments for the challenging and unusual infections caused by *Prototheca*.

## MATERIALS AND METHODS

### Microorganisms

The following *Prototheca* strains were used: *P. bovis* O-2/22 from the outbreak of bovine mastitis (25), *P. bovis* HKmikrobank1 from bovine mastitis (47), *P. blaschkeae* IHEM 26958 from bovine mastitis (48), *P. ciferrii* SAG 2063 from cattle manure (14), and *P. wickerhamii* CCUG 50031 from human skin infection (26). Additionally, *S. aureus* Newman (49), *S. uberis* DSM 20569 (50), *E. coli* ATCC 25922 (51), and *P. aeruginosa* PAO1 (Iglewski) (52) were used. Unless indicated otherwise, microorganisms for experiments were grown overnight at 37°C, on Sabouraud dextrose agar (SDA, Millipore, for algae) or tryptic soy agar (Millipore, for bacteria).

### Naphthoquinones

The following 1,4-naphthoquinones (all from Merck) were used, and prepared as stock solutions in dimethyl sulfoxide (DMSO): 2-methoxy-1,4-naphthoquinone (M-NQ, cat. no. 189162), plumbagin (cat. no. P7262), juglone (cat. no. H47003), lawsone (cat. no. H46805), atovaquone (cat. no. PHR1591), and buparvaquone (cat. no. 32154).

### MIC and MCC measurements

The MIC was measured using the broth microdilution method according to the European Committee on Antimicrobial Susceptibility Testing (EUCAST) yeast guidelines (53). Briefly, RPMI 1640 supplemented with glucose to 2% and buffered with 3-(N-morpholino)propanesulfonic acid (RPMI-2%G) was used as the medium, with a final volume of 200 µL per well of a flat-bottom 96-well polystyrene plate (Sarstedt), with doubling dilutions of the tested drug, inoculated at 0.5–2.5 × 10^5^ CFU/mL in the wells, and incubated at 37°C without agitation for 24 ± 2 h. Drug-free and microorganism-free controls, including added DMSO solvent as needed, were included. Growth was determined by measuring optical density at 530 nm (OD_530_) with FlexStation 3 microplate reader (Molecular Devices), and MIC was defined as the lowest concentration causing ≥90% growth inhibition as compared to the drug-free control.

After MIC measurement, 10 µL from each well was plated on SDA, colonies were counted after 72 h incubation at 37°C, and MCC was defined as the lowest concentration causing ≥99.9% decrease in viability compared to the inoculum.

To ensure uniformity, the above method was used both for the *Prototheca* and for the bacterial strains.

### Growth curves and time-kill curves

*P. bovis* O-2/22 was used to inoculate RPMI-2%G at ≈5 × 10^5^ CFU/mL, with varying concentrations of M-NQ, and was subsequently incubated at 37°C, with shaking. For growth curves, microorganisms were incubated in 96-well plates, 200 µL/well, and at the selected time intervals, the OD_530_ was measured to assess growth. For time-kill curves, microorganisms were incubated in 1.5 mL tubes at 1 mL/tube, and at the selected time intervals, 100 µL samples were drawn from the cultures, and CFU were quantified through serial dilution in phosphate-buffered saline (PBS) and plating on SDA to assess killing. Due to extreme adhesiveness of *P. bovis* to plastic, experiments were performed in Protein LoBind tubes (Eppendorf), and samples were sonicated for 5 min in ultrasonic bath to dislodge microorganisms from the tube walls before CFU enumeration. Notably, the presence of M-NQ on its own had no effect on *P. bovis* adhesion to plastic (Fig. S1). To test the effect of biological fluids, the experiments were conducted like the time-kill curve, but 50% cow milk in PBS (ultra-high-temperature processed, 3.2% fat content, Mlekpol Dairy Cooperative) and 50% human citrated plasma in PBS (Regional Blood Center, Krakow) were inoculated in addition to the RPMI-2%G, and CFU were quantified after 24 h incubation.

### Human cell toxicity assay

Human keratinocyte cell line HaCaT was grown to 75–80% confluency in wells of 96-well cell culture plates in high-glucose DMEM with 10% FBS, at 37°C, 5% CO_2_. Afterward, the medium was exchanged to pure high-glucose DMEM with various concentrations of M-NQ, and after an additional 24 h incubation, cell viability was measured with Calcein AM from LIVE/DEAD Viability/Cytotoxicity Kit for mammalian cells (Thermo Fisher).

### Biofilm destruction assays

To measure the ability of M-NQ to destroy existing biofilms, *P. bovis* O-2/22 was inoculated into 200 µL of RPMI-2%G in wells of a flat-bottom 96-well polystyrene plate (or into 300 µL in a 8-well ibiTreat µSlide, Ibidi, for biofilm microscopy) at a final concentration of ≈1.5 × 10^5^ CFU/mL, and biofilm was allowed to form during 24 h incubation at 37°C. Afterward, the medium was aspirated, biofilm at the bottom of the wells was washed with PBS, and a new medium containing the M-NQ (or DMSO solvent control) was added. After another 24 h of 37°C incubation, the medium was aspirated, the wells were washed with PBS, and biofilms were analyzed in respect to their biomass and microscopic appearance. For biomass quantification, biofilms were fixed by drying the plate at 60°C, followed by staining for 10 min with 0.4% crystal violet, washing with tap water, dissolving the dye bound by biofilm with 33% acetic acid, and measuring absorbance at 595 nm with a microplate reader. Microorganism-free wells were used as blank controls for absorbance reading. For microscopy visualization, biofilms were stained with 10 µM Syto-9 (Invitrogen) in PBS for 30 min at room temperature (a method that reliably stains all microorganisms present in the sample, see Fig. S2), and visualized with Zeiss Axio Observer fluorescence microscope equipped with Zeiss ZEN 3.7 software. Images from five random areas per well were collected, and % area covered by biofilm was automatically counted with FIJI software (54).

### Electron paramagnetic resonance spectroscopy

EPR measurements were carried out using a Bruker EMX-AA EPR spectrometer (Bruker BioSpin) in quartz aqueous flat cell at room temperature. The instrument parameters set were at: center field 3390 G, sweep width 25 G, attenuation 13 dB, microwave power 10.57 mW, receiver gain 7.96 × 10^4^, modulation amplitude 0.5 G, time constant 0.327 s, sweep time 81.92 s, and 60 independent scans for single series measurement. Sample was prepared by combining MN-Q (final concentration 200 µg/mL) and/or *P. bovis* O-2/22 (final OD_600_ = 4) in a total volume of 200 µL of RPMI-2%G, and collecting the EPR spectrum after ≈40 min at room temperature.

Simulation of EPR spectra for M-NQ semiquinone was performed with EasySpin toolbox for MATLAB (55). Spectrum was fitted using simplex and Levenberg-Marquardt approaches and the following parameters of the fit were determined: g-factor (2.0084), hyperfine splitting constants (A_H_ = 1.045 G, A_H_ = 1.368 G, A_H_ = 0.830 G, and A_H_ = 1.226 G), Gaussian peak-to-peak broadening (0.0057 G).

### Biochemical detection of oxidative stress and injury

To measure the level of cellular antioxidant thiols, *P. bovis* O-2/22 was used to inoculate RPMI-2%G at ≈3 × 10^7^ CFU/mL, with 10 µg/mL of M-NQ (or DMSO solvent as a negative control, and 50 nM H_2_O_2_ as a positive control), and was incubated at 37°C, with shaking, for 3 h. Afterward, *Prototheca* was pelleted by centrifugation, homogenized with 1 mm zirconium beads for 15 min at 4°C in a TissueLyser LT (Quiagen) in 5% trichloroacetic acid, centrifuged to remove cell debris, and the extracted total soluble thiols were determined using Ellman’s reagent (22).

Alternatively, to measure oxidation of proteins, coumarin boronic acid (CBA) was used as the sensitive fluorogenic probe as described (56). The cells were incubated with catalase (580 U/mL, 2 min) before homogenization, and homogenization was performed as described above, but in 0.1 mM pentetic acid with 100 U/mL catalase. The fluorescence of 4-hydroxycoumarin formed after the addition of CBA to the protein extract was measured at 10 min intervals with a ClarioStar plate reader (BMG-Labtech) at Ex/Em 360 nm/465 nm for 50 min and *V*_max_ of the reaction was calculated.

To measure the overall redox state of the algae, a 2′,7′-dichlorodihydrofluorescein diacetate (DCFH-DA, Merck) was used, which upon loading into cells is cleaved into DCFH, which in turn could be oxidized into a fluorescent product. *P. bovis* O-2/22 was suspended in distilled water at OD_600_ = 1 and loaded by incubation for 15 min at 37°C with 20 µM DCFH-DA. Afterward, the M-NQ was added to a final concentration of 0.5× MIC, and fluorescence of algal suspension (excitation: 485; emission: 535) was measured in a 96-well black plate (Sarstedt) with a microplate reader every 10 min over 3 h incubation at 37°C. A 50 nM H_2_O_2_ was used as a positive control.

### Mouse *Prototheca* skin infection model

A model combining previously described mouse *Candida* and *S. aureus* skin infection models was used (57, 58). One day before infection, 8- to 9-week-old female C57BL/6 mice (Animal Facility at Faculty of Biochemistry, Biophysics and Biotechnology, Jagiellonian University) had their abdomens shaved and were injected subcutaneously with cortisone acetate at 225 mg/kg body weight to induce a transient immunosuppression. *P. bovis* O-2/22 from overnight culture in yeast-peptone-dextrose broth (YPD, Millipore) was washed and resuspended in PBS at 2 × 10^7^ CFU/mL, and 50 µL of this suspension was injected intradermally through an insulin syringe into abdominal skin. Developing skin changes were scored daily with a 0–2 severity scale (0—no changes; 0.5—red or peeling skin without swelling; 1—small swelling; and 2—large clearly visible swelling below skin surface). On day 3 after injection, mice were euthanized, 8 mm diameter punch biopsies of the infected skin were taken, homogenized with 5 mm stainless steel beads in a TissueLyser LT, and plated on SDA with 50 µg/mL chloramphenicol (preventing skin bacteria growth) to quantify the *Prototheca* load.

Beginning 12 h after infection (when first changes on skin became apparent), and then at 12 h intervals, mice were either treated with M-NQ at 15 mg/kg body weight (suspended in 0.5% sodium carboxymethyl cellulose as a carrier), or with the carrier alone (control group), administered through oral gavage. The dosing was based on a previously reported treatment scheme causing no toxicity in mice (30).

Animal experiments were conducted in the ABSL2 Animal Facility of the Malopolska Center of Biotechnology, Jagiellonian University, and were approved by the 1st Local Institutional Animal Care and Use Committee in Krakow.

### Statistics

All *in vitro* data were collected in a minimum of two independent experiments. For MIC and MCC, the modal values were used. Differences between the treatment groups and the control group were analyzed by ANOVA with Dunnett’s multiple comparison post-test, with *t*-test, or Mann-Whitney test, all with two-tailed *P* values. IC_50_ concentration for human cells was estimated using a four-parameter dose-response curve model. Prism 7 (GraphPad Software) was used for calculations.

## SUPPLEMENTAL MATERIAL

Supplemental material aac.00497-25-s0001.pdf Fig. S1 and S2.

ASM does not own the copyrights to Supplemental Material that may be linked to, or accessed through, an article. The authors have granted ASM a non-exclusive, world-wide license to publish the Supplemental Material files. Please contact the corresponding author directly for reuse.

## References

[1] Kano R. 2020. Emergence of fungal-like organisms: prototheca. Mycopathologia 185:747–754. doi:10.1007/s11046-019-00365-4 31401758

[2] Libisch B, Picot C, Ceballos-Garzon A, Moravkova M, Klimesová M, Telkes G, Chuang S-T, Le Pape P. 2022. Prototheca infections and ecology from a one health perspective. Microorganisms 10:938. doi:10.3390/microorganisms10050938 35630382 PMC9144699

[3] Shave CD, Millyard L, May RC. 2021. Now for something completely different: Prototheca, pathogenic algae. PLoS Pathog 17:e1009362. doi:10.1371/journal.ppat.1009362 33793666 PMC8016101

[4] Ely VL, Espindola JP, Barasuol BM, Sangioni LA, Pereira DB, Botton S de A. 2023. Protothecosis in veterinary medicine: a minireview. Lett Appl Microbiol 76:ovad066. doi:10.1093/lambio/ovad066 37286817

[5] Milanov D, Petrović T, Polaček V, Suvajdžić L, Bojkovski J. 2016. Mastitis associated with Prototheca zopfii - an emerging health and economic problem on dairy farms. J Vet Res 60:373–378. doi:10.1515/jvetres-2016-0054

[6] Huilca-Ibarra MP, Vasco-Julio D, Ledesma Y, Guerrero-Freire S, Zurita J, Castillejo P, Barceló Blasco F, Yanez L, Changoluisa D, Echeverría G, Bastidas-Caldes C, Waard JH de. 2022. High prevalence of prototheca bovis infection in dairy cattle with chronic mastitis in ecuador. Vet Sci 9:659. doi:10.3390/vetsci9120659 36548820 PMC9784310

[7] Jagielski T, Krukowski H, Bochniarz M, Piech T, Roeske K, Bakuła Z, Wlazło Ł, Woch P. 2019. Prevalence of Prototheca spp. on dairy farms in Poland - a cross-country study. Microb Biotechnol 12:556–566. doi:10.1111/1751-7915.13394 30891936 PMC6465227

[8] Park HS, Moon DC, Hyun BH, Lim SK. 2019. Short communication: occurrence and persistence of Prototheca zopfii in dairy herds of Korea. J Dairy Sci 102:2539–2543. doi:10.3168/jds.2018-14979 30612806

[9] Shahid M, Ali T, Zhang L, Hou R, Zhang S, Ding L, Han D, Deng Z, Rahman A, Han B. 2016. Characterization of Prototheca zopfii genotypes isolated from cases of bovine mastitis and cow barns in china. Mycopathologia 181:185–195. doi:10.1007/s11046-015-9951-9 26450620

[10] Jagielski T, Proskurnicka A, Iskra M, Wronka S, Bakuła Z, Danesi P, de Farias MR, Ramos Portilho FV, Garcia Ribeiro M, Rösler U, Kano R, Malik R. 2025. Protothecosis in dogs: a narrative review. J Vet Intern Med 39:e70025. doi:10.1111/jvim.70025 40072265 PMC11898871

[11] Stenner VJ, Mackay B, King T, Barrs VRD, Irwin P, Abraham L, Swift N, Langer N, Bernays M, Hampson E, Martin P, Krockenberger MB, Bosward K, Latter M, Malik R. 2007. Protothecosis in 17 Australian dogs and a review of the canine literature. Med Mycol 45:249–266. doi:10.1080/13693780601187158 17464846

[12] Todd JR, King JW, Oberle A, Matsumoto T, Odaka Y, Fowler M, Pore RS, Shahan TA, Yin L, Sanusi ID. 2012. Protothecosis: report of a case with 20-year follow-up, and review of previously published cases. Med Mycol 50:673–689. doi:10.3109/13693786.2012.677862 22571772

[13] Bozzo G, Bonerba E, Di Pinto A, Bolzoni G, Ceci E, Mottola A, Tantillo G, Terio V. 2014. Occurrence of Prototheca spp. in cow milk samples. New Microbiol 37:459–464. 25387284

[14] Jagielski T, Bakuła Z, Gawor J, Maciszewski K, Kusber W-H, Dyląg M, Nowakowska J, Gromadka R, Karnkowska A. 2019. The genus Prototheca (Trebouxiophyceae, Chlorophyta) revisited: implications from molecular taxonomic studies. Algal Res 43:101639. doi:10.1016/j.algal.2019.101639

[15] McDonald JS, Richard JL, Anderson AJ. 1984. Antimicrobial susceptibility of Prototheca zopfii isolated from bovine intramammary infections. Am J Vet Res 45:1079–1080. 6742568

[16] Proskurnicka A, Żupnik K, Bakuła Z, Iskra M, Rösler U, Jagielski T. 2023. Drug susceptibility profiling of Prototheca species isolated from cases of human protothecosis. Antimicrob Agents Chemother 67:e0162722. doi:10.1128/aac.01627-22 36943065 PMC10112244

[17] Tan K, Huang Z, Ji R, Qiu Y, Wang Z, Liu J. 2019. A review of allelopathy on microalgae. Microbiology (Reading) 165:587–592. doi:10.1099/mic.0.000776 30688632

[18] Wang T, Liu H. 2023. Aquatic plant allelochemicals inhibit the growth of microalgae and cyanobacteria in aquatic environments. Environ Sci Pollut Res 30:105084–105098. doi:10.1007/s11356-023-29994-5 37740161

[19] Meyer GW, Bahamon Naranjo MA, Widhalm JR. 2021. Convergent evolution of plant specialized 1,4-naphthoquinones: metabolism, trafficking, and resistance to their allelopathic effects. J Exp Bot 72:167–176. doi:10.1093/jxb/eraa462 33258472 PMC7853596

[20] Widhalm JR, Rhodes D. 2016. Biosynthesis and molecular actions of specialized 1,4-naphthoquinone natural products produced by horticultural plants. Hortic Res 3:16046. doi:10.1038/hortres.2016.46 27688890 PMC5030760

[21] Kessler CT. 1989. Effect of juglone on freshwater algal growth. J Chem Ecol 15:2127–2134. doi:10.1007/BF01207443 24272301

[22] Nowicka B, Walczak J, Kapsiak M, Barnaś K, Dziuba J, Suchoń A. 2023. Impact of cytotoxic plant naphthoquinones, juglone, plumbagin, lawsone and 2-methoxy-1,4-naphthoquinone, on Chlamydomonas reinhardtii reveals the biochemical mechanism of juglone toxicity by rapid depletion of plastoquinol. Plant Physiol Biochem 197:107660. doi:10.1016/j.plaphy.2023.107660 36996637

[23] Nowicka B, Żądło A, Pluciński B, Kruk J, Kuczyńska P. 2017. The oxidative stress in allelopathy: participation of prenyllipid antioxidants in the response to juglone in Chlamydomonas reinhardtii. Phytochemistry 144:171–179. doi:10.1016/j.phytochem.2017.09.012 28942064

[24] Randall VD, Bragg JD. 1986. Effects of juglone (5’-hydroxy-1, 4-naphthoquinone) on the algae anabaena flos-aquae, nostoc commune, and scenedesmus acuminatus. J Ark Acad Sci 40:52–55.

[25] Beinhauerova M, Moravkova M, Seydlova R, Crhanova M. 2023. Eradication of bovine mastitis caused by the pathogenic microalga prototheca bovis on a dairy cattle farm: a case report. Microbiol Res (Pavia) 14:1343–1352. doi:10.3390/microbiolres14030091

[26] Kwiecinski J. 2015. Biofilm formation by pathogenic Prototheca algae. Lett Appl Microbiol 61:511–517. doi:10.1111/lam.12497 26394169

[27] Nirmal N, Koirala P, Khanashyam AC, Panichayupakaranant P, Septama AW. 2024. Combined effect of Brazilin-rich extract and lawsone methyl ether against infection-causing bacteria. Saudi J Biol Sci 31:103999. doi:10.1016/j.sjbs.2024.103999 38646564 PMC11031759

[28] Sakunphueak A, Panichayupakaranant P. 2012. Comparison of antimicrobial activities of naphthoquinones from Impatiens balsamina. Nat Prod Res 26:1119–1124. doi:10.1080/14786419.2010.551297 21895457

[29] Wang YC, Li WY, Wu DC, Wang JJ, Wu CH, Liao JJ, Lin CK. 2011. In vitro activity of 2-methoxy-1,4-naphthoquinone and stigmasta-7,22-diene-3β-ol from impatiens balsamina L. against multiple antibiotic-resistant Helicobacter pylori. Evid Based Complement Alternat Med 2011:704721. doi:10.1093/ecam/nep147 19773391 PMC3137247

[30] Xu L, Zhou Y, Ou D, Yang H, Feng H, Song H, Xie N, Niu X, Deng X, Sun M, Zhang P, Liu D, Wang J. 2024. Potent synergistic efficacy of 2-methoxy-1,4-naphthoquinone derived from quinones against drug-resistant bacteria. One Health Adv 2:1. doi:10.1186/s44280-023-00030-y

[31] Yang JY, Lee HS. 2015. Antimicrobial activities of active component isolated from Lawsonia inermis leaves and structure-activity relationships of its analogues against food-borne bacteria. J Food Sci Technol 52:2446–2451. doi:10.1007/s13197-013-1245-y 25829631 PMC4375244

[32] Panichayupakaranant P, Reanmongkol W. 2002. Evaluation of chemical stability and skin irritation of lawsone methyl ether in oral base. Pharm Biol 40:429–432. doi:10.1076/phbi.40.6.429.8443

[33] Henning PA, Lee RE. 1976. Effects of selected herbicides and plant hormones on Prototheca wickerhamii. J Med Microbiol 9:23–27. doi:10.1099/00222615-9-1-23 1263245

[34] Casselton PJ. 1966. Further observations on the inhibition of Prototheca zopfii growth by 3-amino-1,2,4-triazole. Physiol Plant 19:411–416.

[35] Siegel JN, Gentile AC. 1966. Effect of 3-amino-1,2,4-triazole on histidine metabolism in algae. Plant Physiol 41:670–672. doi:10.1104/pp.41.4.670 5932405 PMC1086403

[36] Cunha LT, Pugine SMP, Lins PG, Brunetti IL, De Melo MP. 2015. Induction of oxidative stress in Prototheca zopfii by indole-3-acetic acid/HRP or 2,4-pentanedione/HRP systems and their oxidation products. Mycopathologia 179:73–79. doi:10.1007/s11046-014-9807-8 25173924

[37] Pelekis ML, Mangat BS, Krishnan K. 1987. Influence of 2,4-dichlorophenoxyacetic acid on the growth and stored polyglucan content of three species of heterotrophic algae. Pestic Biochem Physiol 28:349–353. doi:10.1016/0048-3575(87)90130-1

[38] Makarova O, Steinke D, Roesler U. 2025. Herbicide glyphosate efficiently inhibits growth of pathogenic Prototheca algae species, suggesting the presence of novel pathways for the development of anti-algal drugs. Microbiol Spectr. doi:10.1128/spectrum.02343-24:e0234324 PMC11878087 39868990

[39] Morello L, Tiroli T, Aretino F, Morandi S, Breviario D. 2020. Preliminary results, perspectives, and proposal for a screening method of in vitro susceptibility of prototheca species to antimicrotubular agents. Antimicrob Agents Chemother 64:e01392-19. doi:10.1128/AAC.01392-19 31871079 PMC7038246

[40] Navarro-Tovar G, Vega-Rodríguez S, Leyva E, Loredo-Carrillo S, de Loera D, López-López LI. 2023. The relevance and insights on 1,4-naphthoquinones as antimicrobial and antitumoral molecules: a systematic review. Pharmaceuticals (Basel) 16:496. doi:10.3390/ph16040496 37111253 PMC10144089

[41] Krylova NG, Kulahava TA, Cheschevik VT, Dremza IK, Semenkova GN, Zavodnik IB. 2016. Redox regulation of mitochondrial functional activity by quinones. Physiol Int 103:439–458. doi:10.1556/2060.103.2016.4.4 28229632

[42] Song R, Yu B, Friedrich D, Li J, Shen H, Krautscheid H, Huang SD, Kim MH. 2020. Naphthoquinone-derivative as a synthetic compound to overcome the antibiotic resistance of methicillin-resistant S. aureus. Commun Biol 3:529. doi:10.1038/s42003-020-01261-0 32973345 PMC7518446

[43] Klotz LO, Hou X, Jacob C. 2014. 1,4-naphthoquinones: from oxidative damage to cellular and inter-cellular signaling. Molecules 19:14902–14918. doi:10.3390/molecules190914902 25232709 PMC6270801

[44] Faizan S, Mohammed Abdo Mohsen M, Amarakanth C, Justin A, Ravishankar Rahangdale R, Raghu Chandrashekar H, Prashantha Kumar BR. 2024. Quinone scaffolds as potential therapeutic anticancer agents: chemistry, mechanism of actions, structure-activity relationships and future perspectives. Results Chem 7:101432. doi:10.1016/j.rechem.2024.101432

[45] Pereyra CE, Dantas RF, Ferreira SB, Gomes LP, Silva-Jr FP. 2019. The diverse mechanisms and anticancer potential of naphthoquinones. Cancer Cell Int 19:207. doi:10.1186/s12935-019-0925-8 31388334 PMC6679553

[46] Ferreira VF, de Carvalho AS, Ferreira PG, Lima CGS, de C da Silva F. 2021. Quinone-based drugs: an important class of molecules in medicinal chemistry. Med Chem 17:1073–1085. doi:10.2174/1573406416666201106104756 33155925

[47] Krukowski H, Lassa H, Zastempowska E, Smulski S, Bis-wencel H. 2020. Etiological agents of bovine mastitis in Poland. Med Weter 76:6339–2020. doi:10.21521/mw.6339

[48] Morandi S, Cremonesi P, Povolo M, Capra E, Silvetti T, Castiglioni B, Ribeiro MG, Alves AC, da Costa GM, Luini M, Brasca M. 2017. Prototheca blaschkeae subsp. brasiliensis subsp. nov., isolated from cow milk. Int J Syst Evol Microbiol 67:3865–3871. doi:10.1099/ijsem.0.002209 28884665

[49] Baba T, Bae T, Schneewind O, Takeuchi F, Hiramatsu K. 2008. Genome sequence of Staphylococcus aureus strain Newman and comparative analysis of staphylococcal genomes: polymorphism and evolution of two major pathogenicity islands. J Bacteriol 190:300–310. doi:10.1128/JB.01000-07 17951380 PMC2223734

[50] Kosecka-Strojek M, Sabat AJ, Akkerboom V, Kooistra-Smid AMDM, Miedzobrodzki J, Friedrich AW. 2019. Development of a reference data set for assigning Streptococcus and Enterococcus species based on next generation sequencing of the 16S-23S rRNA region. Antimicrob Resist Infect Control 8:178. doi:10.1186/s13756-019-0622-3 31788235 PMC6858756

[51] Minogue TD, Daligault HA, Davenport KW, Bishop-Lilly KA, Broomall SM, Bruce DC, Chain PS, Chertkov O, Coyne SR, Freitas T, Frey KG, Gibbons HS, Jaissle J, Redden CL, Rosenzweig CN, Xu Y, Johnson SL. 2014. Complete genome assembly of Escherichia coli ATCC 25922, a serotype O6 reference strain. Genome Announc 2. doi:10.1128/genomeA.00969-14 PMC4175212 25291776

[52] Chandler CE, Horspool AM, Hill PJ, Wozniak DJ, Schertzer JW, Rasko DA, Ernst RK. 2019. Genomic and phenotypic diversity among ten laboratory isolates of Pseudomonas aeruginosa PAO1. J Bacteriol 201:e00595-18. doi:10.1128/JB.00595-18 30530517 PMC6379574

[53] Arendrup MC, Meletiadis J, Mouton JW, Lagrou K, Hamal P, Guinea J, EUCAST-AFST. 2020. EUCAST definitive document E.def 7.3.2: method for the determination of broth dilution minimum inhibitory concentrations of antifungal agents for yeasts 10.1111/j.1469-0691.2012.03880.x 22563750

[54] Schindelin J, Arganda-Carreras I, Frise E, Kaynig V, Longair M, Pietzsch T, Preibisch S, Rueden C, Saalfeld S, Schmid B, Tinevez JY, White DJ, Hartenstein V, Eliceiri K, Tomancak P, Cardona A. 2012. Fiji: an open-source platform for biological-image analysis. Nat Methods 9:676–682. doi:10.1038/nmeth.2019 22743772 PMC3855844

[55] Stoll S, Schweiger A. 2006. EasySpin, a comprehensive software package for spectral simulation and analysis in EPR. J Magn Reson 178:42–55. doi:10.1016/j.jmr.2005.08.013 16188474

[56] Olchawa MM, Szewczyk GM, Zadlo AC, Sarna MW, Wnuk D, Sarna TJ. 2020. The effect of antioxidants on photoreactivity and phototoxic potential of RPE melanolipofuscin granules from human donors of different age. Antioxidants (Basel) 9:1044. doi:10.3390/antiox9111044 33114498 PMC7693403

[57] Conti HR, Huppler AR, Whibley N, Gaffen SL. 2014. Animal models for candidiasis. Curr Protoc Immunol 105:19. doi:10.1002/0471142735.im1906s105 PMC4088949 24700323

[58] Kwiecinski JM, Kratofil RM, Parlet CP, Surewaard BGJ, Kubes P, Horswill AR. 2021. Staphylococcus aureus uses the ArlRS and MgrA cascade to regulate immune evasion during skin infection. Cell Rep 36:109462. doi:10.1016/j.celrep.2021.109462 34320352 PMC8450000

