# Supplementary material for: Allelopathic compound 2-methoxy-1,4-naphthoquinone is broadly effective against pathogenic Prototheca species in vitro and in vivo
Source: Antimicrob Agents Chemother. 2025 Aug 27;69(10):e00497-25. doi: 10.1128/aac.00497-25 (PMC12486852; doi:10.1128/aac.00497-25)
Supplement: Supplemental material — Fig. S1 and S2. [file aac.00497-25-s0001.pdf]

Supplementary Materials

Allelopathic compound 2-methoxy-1,4-naphthoquinone is broadly effective against pathogenic *Prototheca* species *in vitro* and *in vivo*

Amir Aliramezani, Grzegorz Szewczyk, Krystian Mokrzyński, Izabela Ciastoń,

Beatrycze Nowicka, Jakub M. Kwiecinski

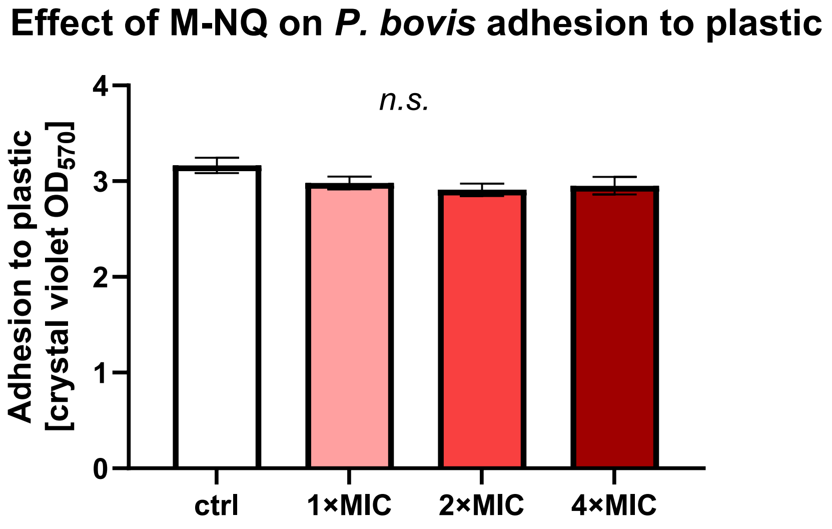

Supplementary Figure 1. M-NQ does not affect adhesion of P. bovis O-2/22 to plastic. *P. bovis* suspended in RPMI-2%G at OD_{530}=1 with or without added M-NQ was incubated in wells of plastic 96-well plate for 1h at 37°C. Afterwards suspensions were aspirated, wells were washed with PBS, *Prototheca* cells remaining attached to wells were stained with crystal violet, which was subsequently dissolved with 33% acetic acid, and absorbance was measured at OD_{570}, corresponding to number of the adhered cells. Statistical significance was determined by the ANOVA with Dunnett's multiple comparison post-test; *n.s.* not significant. Data are shown as means ± SEM; n=10.

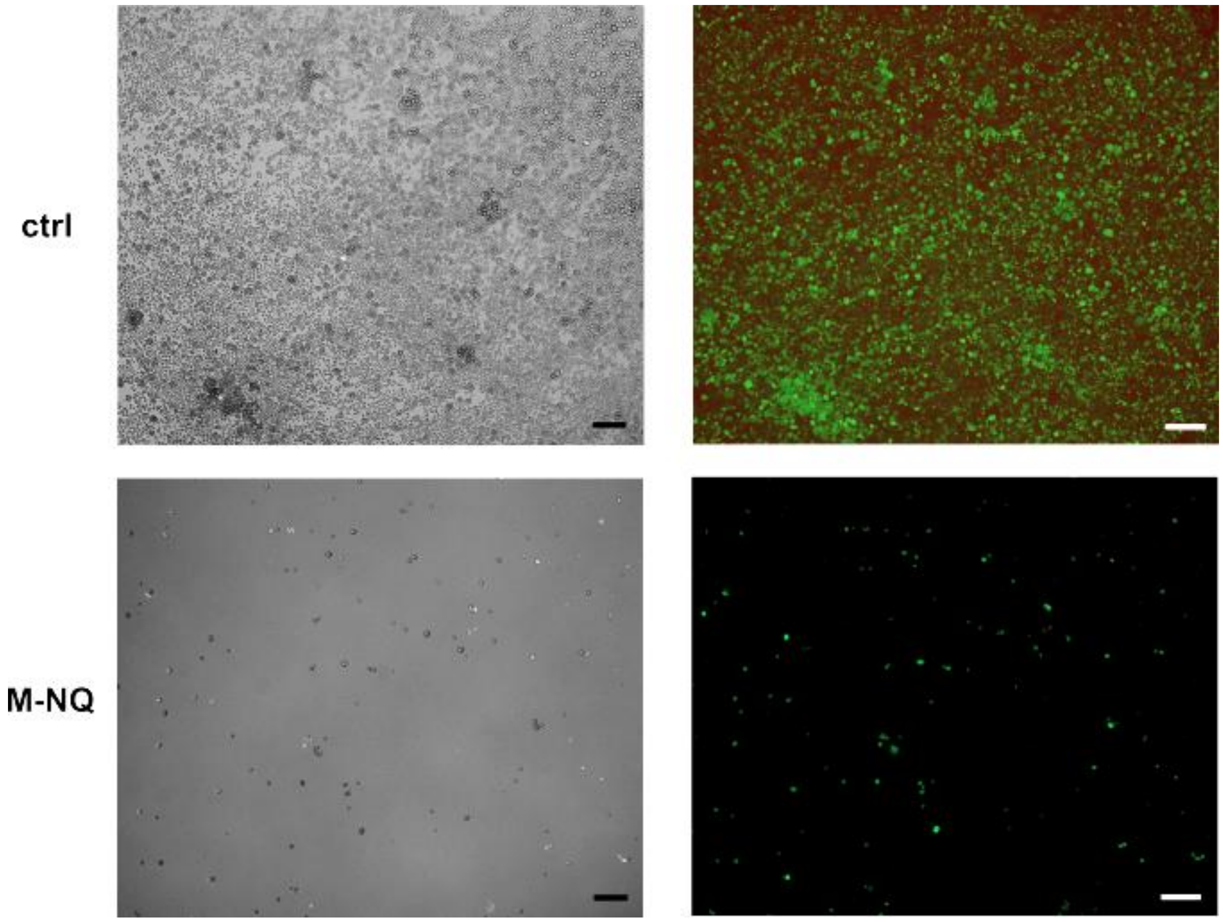

Supplementary Figure 2. Syto 9 dye reliably stains cells in *P. bovis* O-2/22 biofilms. Corresponding microscopy brightfield images and fluorescent images after Syto9 staining of the control and M-NQ treated biofilms are shown. Scale bar = 100 µm.
